# Directional preference for glioblastoma cancer cell membrane encapsulated nanoparticle population: A probabilistic approach for cancer therapeutics

**DOI:** 10.3389/fimmu.2023.1162213

**Published:** 2023-03-29

**Authors:** Saif Khan, Mohd Wajid Ali Khan, Subuhi Sherwani, Sultan Alouffi, Mohammad Jahoor Alam, Khalid Al-Motair, Shahper Khan

**Affiliations:** ^1^ Department of Basic Dental and Medical Sciences, College of Dentistry, University of Ha’il, Ha’il, Saudi Arabia; ^2^ Medical and Diagnostic Research Centre, University of Ha'il, Ha’il, Saudi Arabia; ^3^ Department of Chemistry, College of Sciences, University of Ha’il, Ha’il, Saudi Arabia; ^4^ Department of Biology, College of Sciences, University of Ha’il, Ha’il, Saudi Arabia; ^5^ Department of Clinical Laboratory Sciences, College of Applied Medical Sciences, University of Ha’il, Ha’il, Saudi Arabia; ^6^ Interdisciplinary Nanotechnology Centre, Aligarh Muslim University, Aligarh, India

**Keywords:** glioblastoma, encapsulated nanoparticle, cancer cell membrane, probabilistic model, homotypic binding, human serum albumin nanoparticles

## Abstract

**Background:**

Selective cancer cell recognition is the most challenging objective in the targeted delivery of anti-cancer agents. Extruded specific cancer cell membrane coated nanoparticles, exploiting the potential of homotypic binding along with certain protein-receptor interactions, have recently proven to be the method of choice for targeted delivery of anti-cancer drugs. Prediction of the selective targeting efficiency of the cancer cell membrane encapsulated nanoparticles (CCMEN) is the most critical aspect in selecting this strategy as a method of delivery.

**Materials and methods:**

A probabilistic model based on binding scores and differential expression levels of Glioblastoma cancer cells (GCC) membrane proteins (factors and receptors) was implemented on python 3.9.1. Conditional binding efficiency (CBE) was derived for each combination of protein involved in the interactions. Selective propensities and Odds ratios in favour of cancer cells interactions were determined for all the possible combination of surface proteins for ‘k’ degree of interaction. The model was experimentally validated by two types of Test cultures.

**Results:**

Several Glioblastoma cell surface antigens were identified from literature and databases. Those were screened based on the relevance, availability of expression levels and crystal structure in public databases. High priority eleven surface antigens were selected for probabilistic modelling. A new term, Break-even point (BEP) was defined as a characteristic of the typical cancer cell membrane encapsulated delivery agents. The model predictions lie within ±7% of the experimentally observed values for both experimental test culture types.

**Conclusion:**

The implemented probabilistic model efficiently predicted the directional preference of the exposed nanoparticle coated with cancer cell membrane (in this case GCC membrane). This model, however, is developed and validated for glioblastoma, can be easily tailored for any type of cancer involving CCMEN as delivery agents for potential cancer immunotherapy. This probabilistic model would help in the development of future cancer immunotherapeutic with greater specificity.

## Introduction

Cancer cell targeting is the most critical step towards its successful therapy. Researchers have employed a myriad of techniques to achieve this selective targeting thereby alleviating undesirable consequences (1–4). Recently, biodegradable nanoparticles coated with extruded target cancer cell membranes, were employed to selectively identify target cancer cells. This specificity originates from homotypic binding of differentially expressed extracellular regions of transmembrane proteins (5–7). Glioma cells are well known for differential expression of several membrane associated surface antigens/proteins (8, 9). This characterizes GBM (Glioblastoma multiforme) cell as suitable candidate/s for selective identification built on homotypic interaction of cell surface proteins/antigens (10–13). Nanoparticles coated with extruded cell membranes from glioma cells, harboring similar levels of differentially expressed surface antigens/proteins, was employed by several researchers to selectively target GBM cells (14–17). Glioma cell surface harbors several receptor-factor complexes. These surface receptor-factor couples possess high affinity for each other. These heterotypic surface interactions are critical for progression and/or inhibition of glioma cell proliferation (9). Hetero-complexes of surface-receptor couples are well defined and explored by several studies (8, 10–16). These hetero complexes (involving receptor- factor couple) may offer significant hinderance to the homotypic selective force exploited by the Cancer cell membrane encapsulated nanoparticles (CCMEN) employed for selectively differentiating healthy cells from GBM cells. The magnitude and direction (favour or opposition) of heterotypic interaction play decisive role in determining the selective potential of the CCMEN. The magnitude (or the strength) and direction of the heterotypic interactions depends upon the differential expression pattern of the receptor-factor couples in glioma cells w.r.t healthy cells. Several studies report expression levels of such receptor-factor couples (18–20). Expression levels may also be extracted from databases such as Gene Expression Omnibus (GEO, 21), Human Protein Atlas (HPA, 22) and The Cancer Genome Atlas (TCGA, 23).

Probabilistic models have been developed for patient-specific combination cancer treatments based on sequencing data and functional assay of the drug (24). Other approachesinvolve the application of probabilistic models for the prediction of the metastatic spread of the tumor (25). In the present study we have encoded a probabilistic model in python 3.9.1 based on the binding potential and expression levels of the surface receptors/proteins (SP) on Glioblastoma cancer cells (GCC), Normal healthy cells (NHC) and their corresponding protein Factors (F). The objective of this model is not only to determine the directional preference of the exposed CCMEN population, but also to characterize the CCMEN for a range of degree of interaction of SP. This model, however, is developed and validated for Glioblastoma, can be easily tailored for any type of cancer involving CCMEN as delivery agents.

## Materials and methods

### Expression levels

Normalized surface gene (receptors) and secreted factors (gene) expression levels for healthy normal brain cells were determined from the Human Protein Atlas (HPA) and GEO (Gene Expression Omnibus) datasets. Secreted levels of the IL-13 (Interleukin-13) for normal healthy brain cells was considered as a baseline (since it has the minimum expression level) and all other gene expression levels (factors and receptors) were represented w.r.t to IL-13. Over/repressed levels of genes (receptors and factors) were determined from the GEO series GSE147352. This series includes 85 adult (age range from 22-81 years) grade Glioblastoma tissues, 18 lower grade glioma tissues and 15 normal brain tissues characterized by rRNA-depleted total RNAseq. Only 85 adult grade glioblastoma and 15 normal brain tissues were included in this study. Altered glioblastoma levels of gene expression was represented as folds (**Table 1**) for each gene considered in this study.

**Table 1:** Expression Levels and Glioblastoma fold change.

Receptors				
Receptor Gene	Description	NEX	rNX	FC
*PDGFRA*	Platelet derived growth factor receptor	7.24	72.43	11.1
*TGFB2R*	Transforming Growth Factor beta Type II Receptor	5.21	52.14	3.58
*EGFR*	Epidermal growth factor receptor	4	40	17.19
*Met R.*	Hepatocyte growth receptor	2.7	27	1.95
*IL4R*	Interleukin-4 receptor	4.2	42	2.28
*IL-13R*	Interleukin-13 receptor	7.34	73.43	4.48
*Tfr2*	Transferrin receptor	0.45	4.5	0.41
*Kdr*	Vascular endothelial growth factor receptor	1.96	19.57	2.55
*FGFR1*	Fibroblast growth factor receptor	12.4	124	1.67
*PLAUR*	Urokinase plasminogen activator surface receptor	2.63	26.29	10.24
*ITGA2B*	Integrin	1.69	16.86	0.71
***Factors Gene***	***Factors***	***NEX***	***rNX***	***FC***
*PDGF*	Platelet derived growth factor	12.77	127.71	1.95
*TGF*	Transforming Growth Factor	8.79	87.86	2.51
*EGF*	Epidermal growth factor	0.27	2.71	1.36
*HGF*	Hepatocyte growth factor	1.31	13.14	1.41
*IL-4*	Interleukin-4	0.2	2	1.1
*IL-13*	Interleukin-13	0.1	1	1.9
*Tf*	Transferrin	7.86	78.57	1.17
*VEGF*	Vascular endothelial growth factor	6.09	60.86	10.21
*FGF2*	Fibroblast growth factor	10.16	101.57	1.33
*PLAU*	Urokinase plasminogen activator	0.87	8.71	32.64
*TNC*	Tenascin-C	1.79	17.86	78.92

NEX, Original Normal brain cell expression levels; rNX, Normal brain cell expression levels w.r.t Interlukin-13; FC, Glioblastoma expr levels vs normal brain cell (fold change: The fold change is derived as the ratio of relative rNX (Normal brain cell expression levels w.r.t Interlukin-13 shown in the table) to relative glioblastoma expression levels w.r.t Interlukin-13 not shown in the table but represented as Fold change FC). Relative glioblastoma expression levels w.r.t Interlukin-13 can be obtained simply by dividing FC by rNX.

### Determination of binding efficiency (Intrinsic affinities)

RCSB Protein data bank (PDB) database was used to retrieve three-dimensional structure of surface protein as well as their respective ligand/s. Discovery studio suit was used to optimize the three-dimensional structures. We removed unwanted redundant molecules. All the protein-protein interactions involving native receptor-factor complex are redocking results since these are available in either protein databank or other predicted databases, all receptor-receptor and non-native cross receptor-factor interactions were determined by performing protein-protein docking. Haddock 2.4 online server (https://wenmr.science.uu.nl/haddock2.4/) was used to do docking and to predict the strength of interaction between ligand-protein as well as protein-protein. The best dock was selected that was based on two parameters i.e Haddock score and Z-score. Prodigy webserver (https://wenmr.science.uu.nl/prodigy/) was used to find out binding affinity between ligand-protein as well as protein-protein. The intrinsic binding scores (BS) are reported in database repository RDO_datasets (link provided in Database availability statement)

### Probabilistic model

A probabilistic model was developed to determine the interaction probabilities (and eventually odds ratios) for the receptor (R) surface proteins (SP) and their combinations present on the glioma CCMEN. The interaction probabilities (P) of individual and combinations of SP receptors on CCMEN were determined towards GCC, NHC and F. The model has the following assumptions:

1. A specific SP or their combination undergoes interaction with the SP on any of the following: GCC only, GCC + F, NHC only, NHC + F or F only.
2. A SP or their combination cannot interact with the SP on both GCC and NHC simultaneously.
3. Atleast one SP interacts with any one instance of the SP of the following: GCC only or NHC only or F only or GCC + F or NHC + F.
4. Interaction of the SP or their combination with the secreted “F only” represents no interaction with either GCC or NHC.
5. No Factor-Factor interactions are considered in this study
6. All other interactions were assumed to result in zero net preference to the specific cell or Factor type.

Probabilities were derived from the conditional binding efficiency (CBE) of surface proteins on CCMEN towards the SP on cell type (GCC or NHC) and/or F. CBE were derived from the BS. Since CBE depends upon the protein type and its conditional expression level (Glioblastoma positive: GCC/F or Negative: NHC; **Table 1**). Final CBE values were derived from the following Eq.1


Eq.1 $$CBE={\text{ BS }}^{*}\text{ MIN }(\frac{{\text{SP}}_{NEX\;}\text{ on CCMEN},{\text{ SP}}_{NEX}\text{ on}\frac{\text{GCC}}{\text{NHC}}}{\text{F}}{)}^{*}\text{ MIN }\left(\frac{SP_{FC}\text{ on CCMEN},{\text{ SP}}_{FC}\text{ on}\frac{\text{GCC}}{\text{NHC}}}{\text{F}}\right)$$

Where,

MIN = select the lower value among the choices.

SP*_NEX_* = Surface Protein normal/native expression level

SP*_FC_* = Surface Protein Fold change (Glioblastoma positive: GCC/F or Negative: NHC)

The model assumes that any number of surface protein types on CCMEN may undergo interaction with the SP of GCC/NHC or F. The degree of interaction (*k*) is defined as the number of SP on CCMEN undergoing interaction simultaneously. Probabilities were calculated for degree of interactions ranging from one to *n* (*n* =11) proteins considered in this study (**Table 1**). Except for single protein interaction, several combinations of proteins may be derived depending upon the degree of interaction. The number of possible combinations (*PC*) for a given degree of interaction (*k*) is given by the following equation (Eq.2)


Eq.2 $$PC=\frac{n!}{\left(n-k\right)!\times k!}$$

Where,

*n*! = factorial of total number of protein types considered in the study (11 in this case).

*k*! = factorial of degree of interaction (range from 1 to 11)

**Figure 1:**
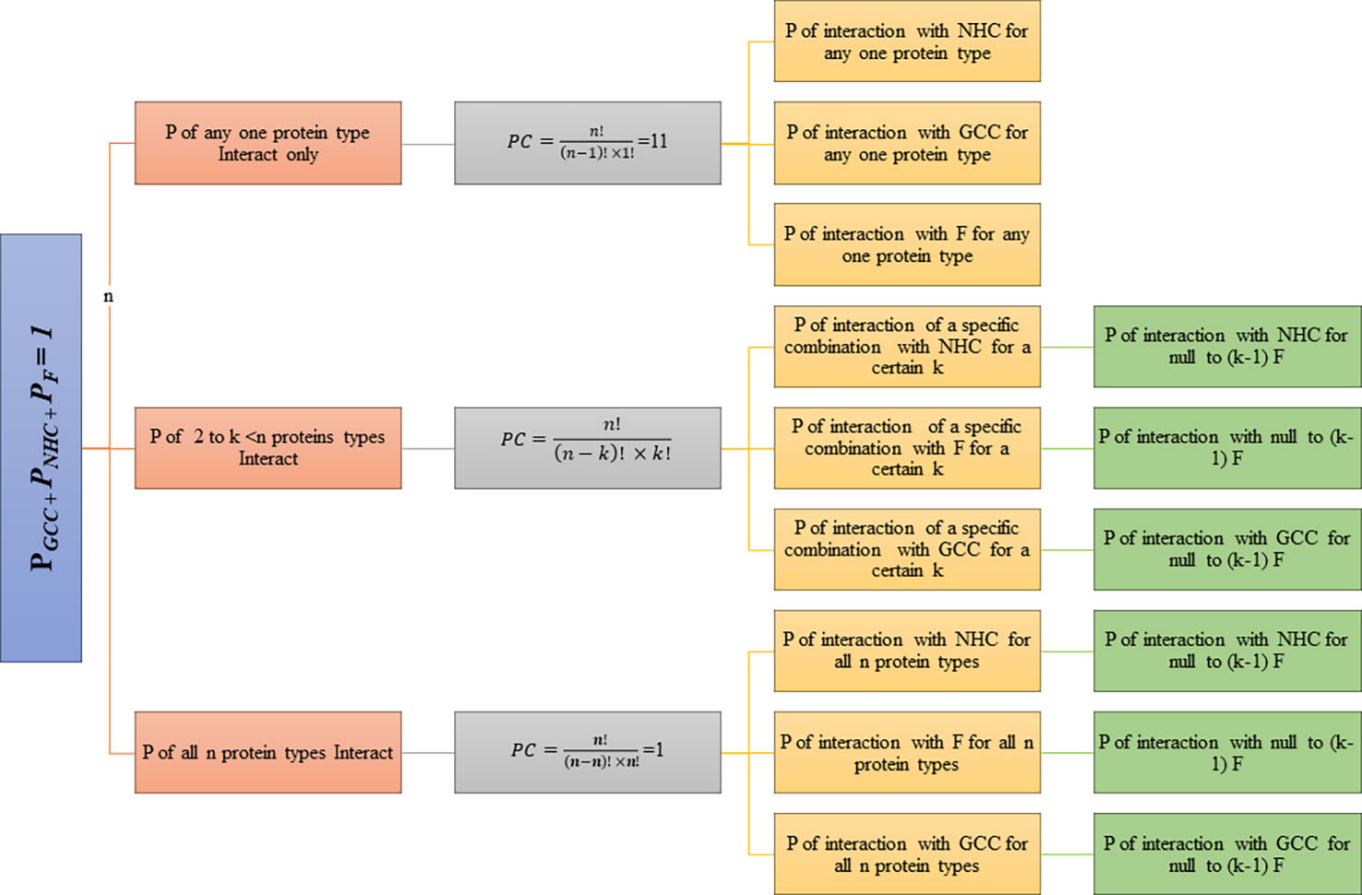
Probabilistic Model.

Probabilities (P) were calculated for each combination assuming null to full factor interactions. The degree of factor interaction (k*_F_*) is defined as the number of factors interacting with a certain combination of degree *k* (0 ≥ k*_F_* ≥ *k*). Binding strengths (BST) and probabilities were calculated for each type of combination. BST*_GCC_* and P*_GCC_* of interaction towards GCC, BST*_NHC_* and P*_NHC_* of interaction towards NHC and BST*_F_* and P*_F_* of interaction towards F. Each combination is further subdivided based on number of factors interacting for a given combination (k*_F_*). The subclasses range from zero F interaction (Full cell type Interaction: GCC/NHC) to full F Interaction (zero cell type Interaction: F). BST and P of interaction was determined for each class and its subdivision. The hierarchy of probabilistic model classes and subclasses is represented in the flowchart (**Figure 1**).

(P*_GCC_*: probability of Interaction with GCC, P*_NHC_*: probability of Interaction with NHC, P*_F_*: probability of Interaction with F, *n*=11)

The model was implemented in python (3.9.1).

### Conditional expected value calculation

Expected value (*E*(*x*)) of a random variable *x* is defined as the sum of the probability-weighted average of all the possible realization of the discreet variable. The Expected value is the arithmetic mean of several independently selected outcomes. In this case the expected values of binding strength were determined (from the probabilistic model discussed above) for each class (*k*) and subclass (k*_F_*) based on their respective conditional probabilities (Conditional probability is defined as the likelihood of an event or outcome occurring, based on the occurrence of a previous event or outcome, in this case depending class: *k* and corresponding subclass: k*_F_*)


$$E{\left(x\right)}_{k,k_{F}}=\;\sum_{i=1}^{i=\frac{n!}{\left(n-k\right)!\times k!}}P_{k,k_{F}}^{BST_{i}^{k,k_{F}}}\times BST_{i}^{k,k_{F}}$$

Here:*E*(x)*_k,kF_* is the expected value of the k^th^ class and k_F_ subclass; *n*=11.

$P_{k,k_{F}}^{BST_{i}^{k,k_{F}}}$ is the conditional probability value of the i^th^ combination for the k^th^ class and subclass k_F_.

$BST_{i}^{k,k_{F}}$ is the binding strength of the individual or specific combination of SP for the k^th^ class and subclass k_F_.

### Determination of selective propensities

**Figure 2:**
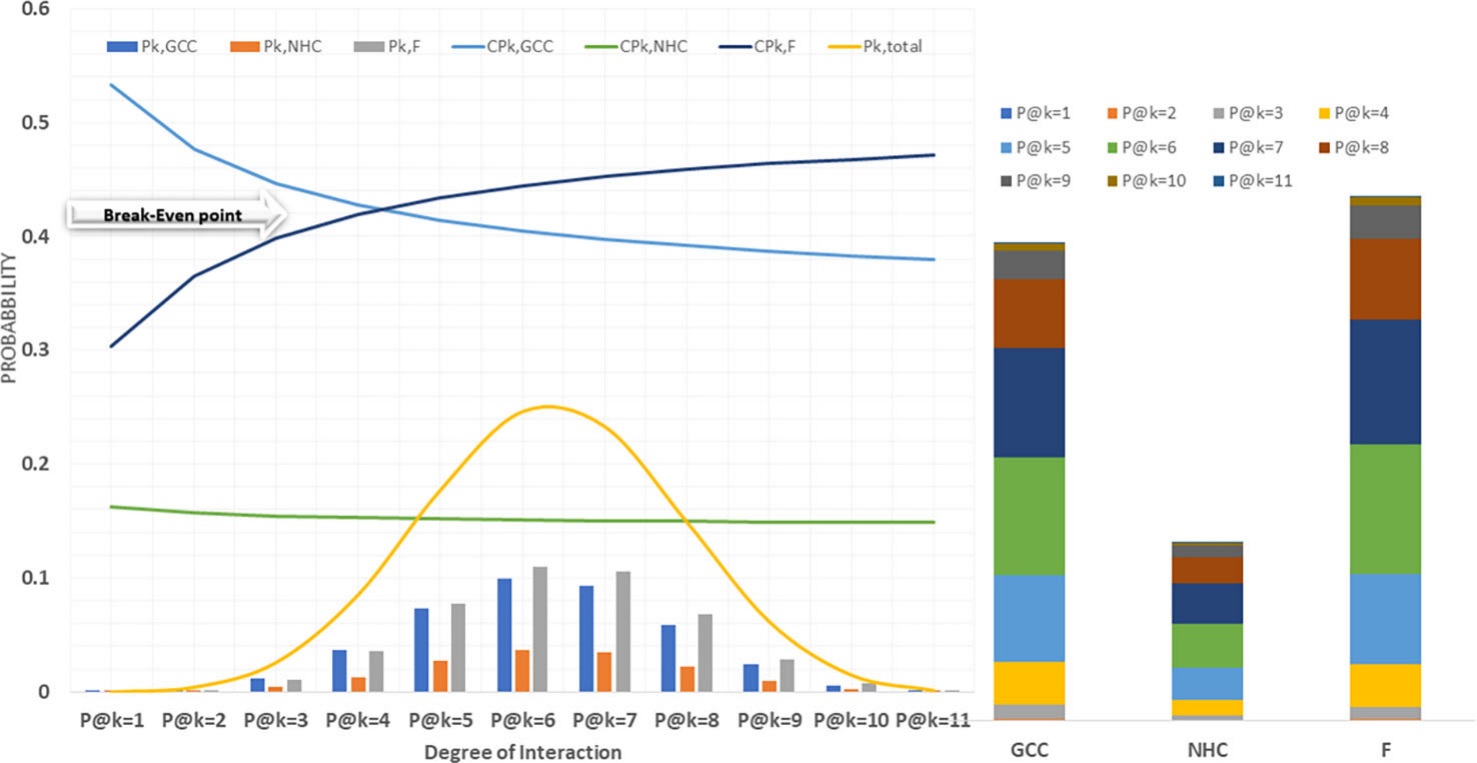
Probability distribution @k interactions; P:Probability, CP: conditional probability.


Selective propensity (*S_p_*) is defined as the affinity of the SP on CCMEN or their combination for a particular degree of protein interaction (*k* and subclass k_F_) towards SP on GCC, NHC or F. *S_p_* for a typical SP combination (for a specific *k* and k_F_) were derived by representing the BSTs (for GCC, NHC and F) as vectors on the *x*, *y* and *z* axis in a three-dimensional space (**Figure 2**). *S_p, GCC/NHC/F_*: selective propensity towards GCC/NHC/F is given by Eq.3-5

Eq.3 $$S_{p,\;GCC}=90{}^{\circ}-R{{}^{\circ}}_{x,\;GCC}$$

Here $:R{{}^{\circ}}_{x}=Angle\;of\;the\;resultant\;vector\;with\;x-axis$ , *x-axis* represents GCC


Eq.4 $$S_{p,\;NHC}=90{}^{\circ}-R{{}^{\circ}}_{x,\;NHC}$$

Here $:R{{}^{\circ}}_{x}=Angle\;of\;the\;resultant\;vector\;with\;x-axis$ , *x-axis* represents NHC


Eq.5 $$S_{p,\;F}=90{}^{\circ}-R{{}^{\circ}}_{x,\;F}$$

Here $:R{{}^{\circ}}_{x}=Angle\;of\;the\;resultant\;vector\;with\;x-axis$ , *x-axis* represents F

$R{{}^{\circ}}_{x,\;GCC/NHC/F}$ was determined as the ratio of the resultant vector *R_x_*, (calculated by Eq.6-11) with the BST for GCC/NHC/F on the *x-axis* (*x-axis* was cycled through BSTs for GCC, NHC and F sequentially)


Eq.6 $$R_{x,\;GCC}=\sqrt[{2}]{BST_{x,GCC}^{2}+\;BST_{y,NHC}^{2}+BST_{z,F}^{2}}$$

Eq.7 $$R_{x,\;NHC}=\sqrt[{2}]{BST_{x,NHC}^{2}+\;BST_{y,GCC}^{2}+BST_{z,F}^{2}}$$

Eq.8 $$R_{x,\;F}=\sqrt[{2}]{BST_{x,F}^{2}+\;BST_{y,GCC}^{2}+BST_{z,NHC}^{2}}$$

Eq.9 $$R{{}^{\circ}}_{x,\;GCC}=Degrees\left(\frac{BST_{x,GCC}}{R_{x,\;GCC}}\right)$$

Eq.10 $$R{{}^{\circ}}_{x,\;NHC}=Degrees\left(\frac{BST_{x,NHC}}{R_{x,\;NCC}}\right)$$

Eq.11 $$R{{}^{\circ}}_{x,\;F}=Degrees\left(\frac{BST_{x,F}}{R_{x,\;F}}\right)$$

*S_p_* is directly proportional to the pull of the SP or their combination towards the axis of interest (*x-axis*). Selective propensities (*S_p_*) of each protein type or their combination for a particular degree of interaction (*k* and for their subclasses k*_F_*) were derived from Eq.3-5.

### Experimental validation of model predictions

An *In vitro* validation experiment was designed to determine the accuracy of the probabilistic model prediction for GCC binding efficiency of the exposed CCMEN population. The initial (exposed) and final concentration of the CCMEN was determined in terms of Na^+^/K^+^ ATPase α-1 cell surface receptor concentration (µg/mL). This receptor is present on the glioblastoma cell membrane encapsulating the HSA-Nanoparticle (described in the database repository RDO_datasets). The concentration was determined by direct binding ELISA for Na^+^/K^+^ ATPase α-1 cell surface receptor.

Standard plot for Na+/K+ ATPase α-1 is given in database repository RDO_datasets: Standard Plot. The *ln* fitting equation (Eq.12) with R² = 0.9158 is given as


Eq.12 Absorbance (nm) = 0.2657ln(Na+/K+ ATPase α−1 µg/mL) + 1.072

Overnight culture of Glioblastoma cancer cells (GCC cells) 10,000 (U87MG) or both: GCC cells and peripheral blood mononuclear cells (PBMCs) in a ratio of 1:1 (5000:5000) were prepared separately in a total volume of 200 μl of complete RPMI medium. CCMEN (5 µg/ml) was added to the Test cultures: Test culture I: GCC + F (released from GCC cells) and Test culture II: GCC + NHC + F (released from GCC and NHC cells) and incubated for 30 mins (duration standardized previously data not provided here). The contents of the test culture were centrifuged after incubation to separate the supernatant for Test culture I and Test culture II. The supernatant was subjected to direct binding ELISA for Na+/K+ ATPase α-1 cell surface receptor (refer to database repository RDO_datasets for details). The fraction of CCMEN population binding the GCC was determined from Eq. 13.


Eq.13 % of CCMEN bound to GCC=conc. of CCMENexposed (µgmL)− conc. of CCMENsupernatant I or II (µgmL)conc. of CCMENexposed (µgmL)x 100

Note:- All the experiments were performed in triplicate and average value were reported.

### Preparation of CCMEN (CCM-c/m-HSA-Cis NPs)

Synthesize of CCMEN was carried out using a previously reported extrusion approach with slight modifications (6, 26). First, cell membrane was isolated from a glioblastoma cancer cell line U87MG. Second, a dual delivery mode HSA NPs was synthesised using cationic HSA (c-HSA) and manno-pyranoside HSA (m-HSA). Both c-HSA (5 mg; 10%) and m-HSA (10 mg; 20%) were mixed together using distilled water. Five gram of cisplatin (free base form) was added to 50 ml of distilled water, and then this was added to the above mixture of albumin in a ratio of 1% w/v in total 10 ml. The mixture will be emulsified using centrifugation at 10,000 rpm for 2 minutes. The pellet was collected and homogenized using a glass homogenizer resulting primary emulsion. Furthermore, a high-pressure homogenizer was used and primary emulsion was passed through (7-9 times) at a pressure of 20,000 psi to get c/m-HSA-Cis NPs. c/m-HSA-Cis NPs were passed through a 200 nm filter. Filtered c/m-HSA-Cis NPs were stored at −80°C for future experiments. Finally, isolated cancer cell membrane (CCM) was 10-15 times extruded physically using a 400 nm polycarbonate membrane and the resulting vesicles were mixed with c/m-HSA-Cis NPs. The mixture was further extruded through a 200 nm polycarbonate membrane to get the final product CCM-c/m-HSA-Cis NPs.

## Results

A probabilistic model was implemented in python to predict the fraction of CCMEN directed towards specific cell type (GCC/NHC) or protein F. Both tabular and graphical results were generated from this tool. The tabular results were presented as.csv files. Several types of.csv files were generated. BST_k_GCC/NHC/F.csv files represent the calculated binding strength for all the possible combinations of the SP of CCMEN towards SP of GCC/NHC/F for *k* degree of interaction (1≤*k ≤* 11). Column headers: Prot_comb represents the receptor numeric code for the specific protein combination (refer to **Table 1** for numeric receptor code: R_code), BST@kf = N is the binding strength for N factor interaction (0≤N≤k). BST@k_interactions.csv file tabulates the overall binding strengths (summation of BST for all combinations) of SP on CCMEN for *k* interaction (1≤*k ≤* 11) towards GCC (first row), NHC (second row) and Factors (third row). P_k_GCC/NHC/F.csv tabulate the absolute probability of interaction derived from the probabilistic model for all the possible combinations of the SP of CCMEN towards SP of GCC/NHC/F for *k* degree of interaction (1≤*k ≤* 11). Column header: P@kf = N is the probability of binding of the specific protein combination (refer to **Table 1** for numeric receptor code: R_code) for N factor interaction (0≤N≤*k*). P@K_interactions.csv file tabulates the overall absolute probabilities of binding (summation of probabilities for all combinations) of SP on CCMEN for *k* interaction (1≤*k ≤* 11) towards GCC (first row), NHC (second row) and Factors (third row). The distribution of overall binding probabilities as a function of 1≤*k ≤* 11 is given in **Figure 2**. SP_k_GCC/NHC/F.csv tabulate the selective propensity (as discussed in methods section) of all the possible combinations of the SP of CCMEN towards SP of GCC/NHC/F for *k* degree of interaction (1≤*k ≤* 11). Column headers: Sp_,prot_comb represents the average selective propensity of the specific SP combination towards GCC/NHC/F, Sp@kf = N is the selective propensity of the specific SP combination for N factor interactions. SP@K_interactions.csv file tabulates the overall selective propensity of binding (summation of selective propensities for all combinations from BST@*k*_interactions.csv) for SP on CCMEN@*k* interaction (1≥*k ≤* 11) towards GCC (first row), NHC (second row) and Factors (third row).

EBST_k_GCC/NHC/F.csv files reports the probabilistic Expected Value *E*(*x*)*_k,kF_* for *BST^k,kF^* (binding strength for *k* interaction and k_F_ factor interactions) towards GCC/NHC/F. Column headers: EBST_Overall is the overall probabilistic *E*(*x*)*_k_* of binding strength for k degree of interaction towards GCC/NHC/F, EBST@kf=N is the probabilistic *E*(*x*)*_k,kF_* of binding strength for *k* degree of interaction and N Factor interactions towards GCC/NHC/F. EBST_k_interactions.csv summarizes the *E*(*x*)*_k_* of BST for GCC, NHC and F for 1≤*k*≤*n*. ESP_k_GCC/NHC/F.csv files tabulates the probabilistic *E*(*x*)*_k,kF_* of selective propensity towards GCC/NHC/F. Column headers: EBST_Overall is the overall probabilistic *E*(*x*)*_k_* of *S_p_* for *k* degree of interaction towards GCC/NHC/F, EBST@kf=N is the probabilistic *E*(*x*)*_k,kF_* of *S_p_* for *k* degree of interaction and N Factor interactions towards GCC/NHC/F. ESP_k_interactions.csv summarizes the *E*(*x*)*_k_* of *S_p_* for GCC, NHC and F for 1≤*k*≤*n*. *E*(*x*)*_k_* results for BST and *S_p_* are reported in database repository RDO_datasets (link provided in Database availability statement).

**Figure 3:**
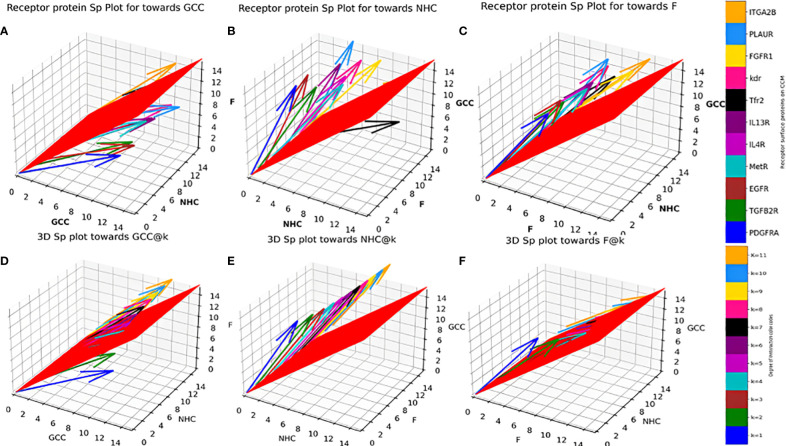
Individual Protein vs Degree of interaction quiver plots; 3D S_p plot for Individual surface proteins towards **(A)** GCC, **(B)** NHC and **(C)** F; 3D S_p plot @k interactions towards **(D)** GCC, **(E)** NHC and **(F)** F.

**Figure 4:**
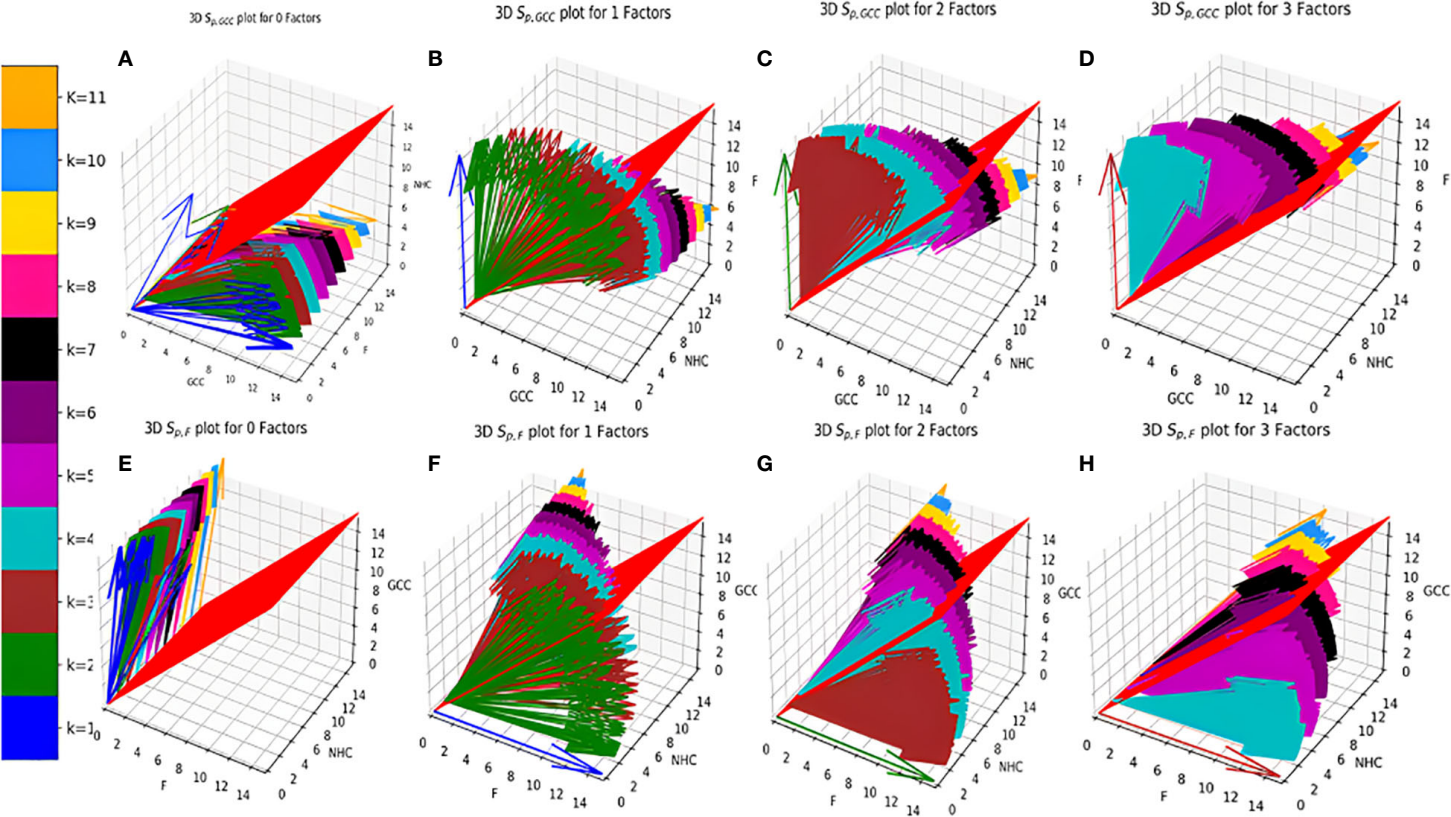
Distribution of $S_{p,GCC}^{k,k_{F}}$ Vs $S_{p,F}^{k,k_{F}}$ **(A–D)**. $S_{p,GCC}^{k,k_{F}}$ **(E–H)**. $S_{p,F}^{k,k_{F}}$ . **(A)** zero Factors; **(B)** 1 Factor; **(C)** 2 Factors; **(D)** 3 Factors; **(E)** zero Factors; **(F)** 1 Factor; **(G)** 2 Factors; **(H)** 3 Factors.

*S_p_* for individual surface receptor protein (**Table 1**) on CCMEN were determined as function of their average respective CBEs towards GCC, NHC and F (Eq. 3-11), **Figures 3A–C** presents the unscaled *S_p_*,*_GCC_*, *S_p_*,*_NHC_* and *S_p_*,*_F_* of individual surface receptor protein as vectors in a 3D interstitial space. The 2D red plane represents threshold boundary below which the arrows parallel to *x*-axis represents the individual protein directed towards cell type/proteins on the *x*-axis. The labels on the three axis *(x, y and z)* represents arbitrary positional coordinates within the interstitial space. $E{\left(x\right)}_{k}$ $S_{p,NHC}^{k}$ and $S_{p,F}^{k}$ were determined for all possible *k* interactions (1≤*k*≤*n*=11; **Figures 3D–F**, File: SP@K_interactions.csv). $S_{p,GCC}^{k,k_{F}}$ and $S_{p,F}^{k,k_{F}}$ were determined for all possible *k* interactions (1≤*k*≤*n*=11) and factor interactions *k_F_* (0≤*k_F_* ≤11). **Figure 4** presents the distribution for the $S_{p,GCC}^{k,k_{F}}$ in contrast to $S_{p,F}^{k,k_{F}}$ up till three factor interactions (rest are provided in database repository RDO_datasets). 3D quiver plots were also determined for $S_{p,NHC}^{k,k_{F}}$ and reported in in database repository RDO_datasets.

**Figure 5:**
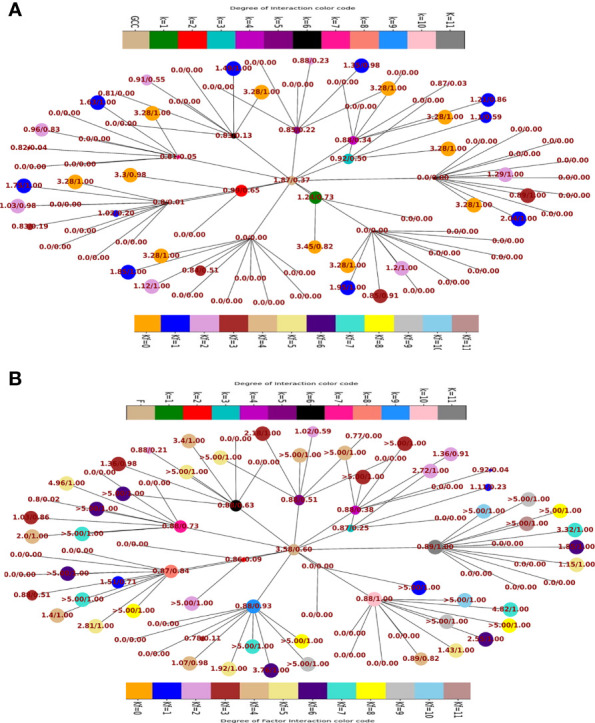
**(A)** Network plot of class wise fractional distribution of $S_{p,GCC}^{k,k_{F}}$ and corresponding odds ratio: node label = odds ratio of the fractional population in favour GCC interaction for the specific class ‘*k’* and subclass ‘*k_F_*’/fraction of population directed towards GCC for the specific class ‘*k’* and subclass ‘*k_F_*’. **(B)** Network plot of class wise fractional distribution of $S_{p,GCC}^{k,k_{F}}$ and corresponding odds ratio: node label = odds ratio of the fractional population in favour of F interaction for the specific class ‘*k’* and subclass ‘*k_F_*’/fraction of population directed towards F for the specific class ‘*k’* and subclass ‘*k_F_*’.

**Figure 6:**
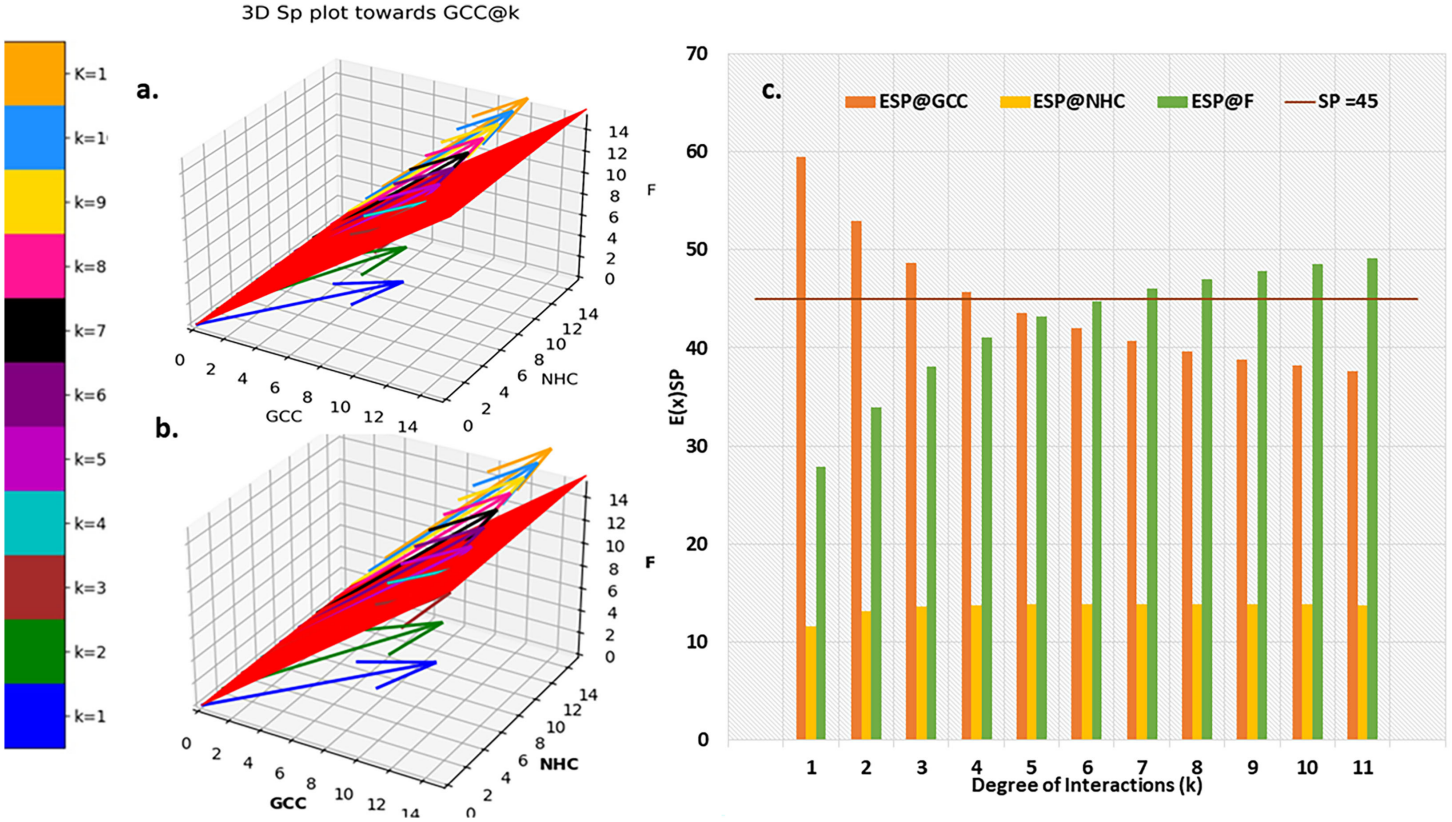
Probabilistic Expected Directional preference. **(A)** 3D *S_p_* plot @*k* interactions towards **(B)** Resultant *E(x)_k_* of *S_p,k_* **(C)** comparison of *E(x)_k_* of *S_p,k_* for GCC, NHC and F.

Fraction of population having $S_{p,GCC}^{k,k_{F}}$ and $S_{p,F}^{k,k_{F}}$ > 45° and the respective odds ratios in favour of GCC (**Figure 5A**) and F (**Figure 5B**) of the fractional population were determined for all classes of interaction level (1≤*k*≥11) and their corresponding subclass (leaves of network) of factor interaction (0≤k_F_ ≤ 11). This was reported as Kamada-kawai network plots (**Figures 5A,B**). The size of nodes corresponds to the fractional population of CCMEN encapsulated nanoparticle (directed towards GCC: **Figure 5A** and towards F: **Figure 5B**) for the specific class ‘*k*’ (Intermediary nodes; refer to color codes in the figure for specific degree of interaction) and/or subclass ‘*k_F_*’ (leaf nodes; refer to color codes in the figure for specific degree of factor interaction). Each node label (**Figure 5A**) = odds ratio of the fractional population in favour GCC interaction for the specific class ‘*k’* and subclass ‘*k_F_*’/fraction of population directed towards GCC for the specific class ‘*k’* and subclass ‘*k_F_*’. Each node label (**Figure 5B**) = odds ratio of the fractional population in favour of F interaction for the specific class ‘*k’* and subclass ‘*k_F_*’/fraction of population directed towards F for the specific class ‘*k’* and subclass ‘*k_F_*’. Kamada-kawai network plots for $S_{p,NHC}^{k,k_{F}}$ > 45° and the respective odds ratios in favour of NHC of the fractional population of CCMEN encapsulated nanoparticle directed towards NHC are provided as in database repository RDO_datasets. *E*(*x*)*_k_*,*s_p_* and *E*(*x*)*_k_*,*s_p_* for a particular *k* degree of interactions are given in **Figure 6**. The horizontal bar for *E*(*x*)*_k_*,*s_p_* represents the threshold (*E*(*x*)*_k_*,*s_p_* ≥ *s_p_* =45°) required propensity to be directed towards *x*-axis (cycled through GCC, NHC and F).

The distribution of resultant *E(x)_k_* of *S_p,k_* towards GCC is portrayed in **Figure 6C**. resultant *E(x)_k_* profile of *S_p,k_* towards NHC and F are provided in in database repository RDO_datasets. Ultimate directional preference (GCC, NHC, F and NNDP) of the CCMEN (**Figure 5A, B**). NNDP corresponds to the fraction of nanoparticles having no net directional preference (NNDP) and hence their fate is unpredictable. The GCC, NHC and F fraction of nanoparticle have a net directional preference towards the GCC, NHC and F respectively.

## Discussion

This study and associated python tool were designed to determine the fraction of CCMEN directed towards the cancer cell (in this case GCC, NHC and extracellular Factors F). This was derived by implementing a probabilistic model (discussed in the methods section) based on the CBE of the of surface receptor proteins on CCMEN for GCC, NHC and F. Eq. 1 explains the derivation of CBE from BS. BS is multiplied with the expression fold change of the contributing (R-R or R-F) proteins having the lower fold change of the two interacting proteins. This magnify/reduce the BS as a function of expression level simultaneously keeping it within the bounds defined by the lower expression fold change. Three types of CBEs were derived: CBE for interaction of SP on CCMEN and GCC (input file: cmcc.csv), CBE for interaction of SP on CCMEN and NHC (input file: cmnc.csv) and CBE for interaction of SP on CCMEN and F (input file: cmf.csv). The probabilistic model reveals the BST and corresponding probabilities of all the possible ways (combinations) the CCMEN may interact with the GCC, NHC and/or F (model assumptions discussed before). **Figure 2** highlight the probability distribution profile of CCMEN interaction with GCC, NHC and F. The distribution is mildly left skewed normal with peak at *k*=6. More than 80% of the exposed CCMEN population have protein interactions ranging from 5 to 8 surface receptors. It is interesting to note that the conditional probability (CP_k_) for interaction of SP on CCMEN with GCC follows an inverse relationship with *k* (**Figure 2**). CP_k_ corresponds to the fractional population of CCMEN for a specific *k*. For *k*≥5 the fractional population directed towards Factors dominates over the fractional population targeting GCC. This point of intersection defined here as the Break-even point (BEP) corresponds to the first instance of CP_k,GCC_ falling below either of the other two (CP_k, NHC_ or CP_k, F_). Higher is the value of BEP more is the fraction of CCMEN directed towards the cancer cells (in this case GCC). BEP ranges from 0 to *n*. Hence, BEP is hereby suggested as a critical scale for measurement of cancer cell targeting efficiency of CCMEN for given surface antigens (receptors).

Individual *S_p_* for all the surface receptor proteins (except Tfr2 and ITGA2B, **Table 1**) on CCMEN are directed towards GCC. (Tfr2: towards NHC, and ITGA2B: no net directional preference, **Figure 3A-C**). However, a sharp contrast is observed in the directional preference of the CCMEN population obtained as a consequence of the real probabilistic picture (based on all possible combinations) from the probabilistic model. The dominant GCC directional preference of CCMEN fades away gradually when the *k*≥5 (**Figures 3D–F**). This may be accounted to the proportionally growing population of CCMEN interacting with the factors, with increasing “*k*”. A detailed picture of the distribution of $S_{p,GCC}^{k,k_{F}}$ (**Figures 4A–D**) and $S_{p,F}^{k,k_{F}}$ (**Figures 4E–H**) reveals the effect of increasing factor interaction for all degrees of *k* (1≥*k ≤* 11, *k_F_ ≤* 3, distribution for *k_F_* ≥4 available in database repository RDO_datasets. It is obvious, that higher factor interactions are only possible at higher *k* values. It is important to note that the increase in the fractional population of CCMEN directed towards F (with increasing *k_F_*) does not exactly correspond to the fractional loss of CCMEN population directed towards GCC. This fractional loss is distributed into three types of CCMEN population having different directional preferences. Type1: Directed towards F; Type2: Directed towards NHC (negligible in this case) and Type3: No Net directional preference (NNDP). CCMEN classified under NNDP category have almost equal propensities towards the three: GCC, NHC and F. $S_{p,GCC}^{k,k_{F}}$ , $S_{p,F}^{k,k_{F}}$ and $S_{p,NHC}^{k,k_{F}}$ for CCMENs under NNDP are always < than the threshold 45° (red 2D plane) for all *k* and *k_F_* values. As per the assumption no. 3 (refer to methods section: probabilistic model assumptions) NNDP population may be equally distributed among the three fates: GCC, NHC and F. In this case, NNDP population corresponds to 3% of the exposed CCMEN. GCC, NHC and F each will get an equal share of ~ 1%. This will be added up to the respective fractional population directed towards GCC, NHC and F derived from the model.

Kamada-kawai network plots (**Figures 5A,B**) reveal the fractional selective preference of each class (*k*: Intermediary nodes) and its corresponding subclass (*k_F_*: Leaf Nodes). No direct proportionality is observed between odds ratio in favour of GCC and F interaction (nodes label: numerator; **Figures 5A, B** respectively) and fraction of population directed towards GCC and F (node labels: denominator; **Figures 5A, B** respectively). The fraction of CCMEN population directed towards GCC falls sharply as the degree of interaction increases. The odds ratio falls below 1 for *k_F_*_≥_3 (for all *k*). The resultant conditional expected directional preference (*E(x)_k_* of *S_p,k_* (refer to Methods section) for GCC (**Figure 6B**) exactly resemble the gross directional preference (**Figure 6A**). *E(x)_k_* of *S_p,k_* for F increases gradually (as ‘k’ tends to 11) and jumps over the threshold (*s_p_* =45°) for k≥6 (**Figure 6C**). Since the *E(x)_5_* of *S_p,5_* for GCC, NHC and F falls short of the threshold this class (*k* =5) may be designated as the class with the highest NNDP population (**Figure 6C**). The overall odds ratio in favour of GCC interaction is almost double (1.87) for a CCMEN fractional population of 37% (directed towards GCC). This odds ratio prescribes a good fidelity of the selective preference of this fraction of population towards GCC. This population primarily corresponds to subclass 0≥*k_F_* < 4 (**Figure 5A**). 60% of the total exposed population of CCMEN is directed towards F with significantly high odds in favour of F interaction (3.58). This fraction of population primarily corresponds to subclass 5≥*k_F_* < 11 (**Figure 5B**). NHC appears to attract a negligible fraction of population (provided in database repository RDO_datasets). The present model has the following shortcomings, this model considers only three type of interactions **i.**e to GCC, NHC and Factors however in realistic scenarios there will be more interactions such as with certain immuno/inflammatory markers. The upcoming upgraded model will be more comprehensive and will include case specific interactions in addition to the present three dimensional. The upcoming model will be able to handle customised N-dimensional interactions. This model does not include Factor-Factor interactions. The upgraded model will be able to handle this type of interactions as well. As and when the model becomes more comprehensive in upcoming upgraded version it will be able to determine the net directional preference of more and more particle fraction thereby reducing the particles within NNDP fraction. The final prediction of the exposed CCMEN population (for Test culture type II, **Figure 5A**): GCC = 38% (37_P_model_ + 1_NNDP_)@Odds ratio in favour of GCC=1.87, NHC = 1% (0 _P_model_ + 1_NNDP_))@Odds ratio in favour of NHC=1.5 and F = 61% (60_P_model_ + 1_NNDP_))@Odds ratio in favour of F=3.58. The model prediction for percentage/fraction of CCMEN (exposed) population directed towards GCC (for Test culture type I): GCC = 39% (39_P_model_ + 0_NNDP_))@Odds ratio in favour of GCC=4.63, NHC = Absent in Test culture type I and F = 61% (61_P_model_ + 0_NNDP_)@Odds ratio in favour of F >5. Model predictions for test culture I were derived by supplying a zero matrix for NHC input file of CBE values (cmnc.csv). The fraction of CCMEN population directed towards GCC, F and NHC were determined experimentally as discussed in methods section (Experimental validation of model predictions: Eq 13). The model predictions lie within ±7% of the experimentally observed values for both (Test culture I and II). The probabilistic model efficiently predicts the directional preference of the nanoparticle population.

## Conclusion

A probabilistic model based on binding scores and expression levels was implemented on python 3.9.1. The implemented probabilistic model efficiently predicted the directional preference (39%) of the exposed CCMEN towards Glioblastoma cancer cells. It is recommended to selectively include those surface antigen on the membrane encapsulated nanoparticles which enhance the value of BEP. Higher the value of BEP more is the fraction of CCMEN directed towards the Cancer cells. Present model may be applied to determine the directional preference of an entity (e.g Nanoparticle coated with cancer cell membrane) under the influence of three directional forces in three dimensions. However, upcoming versions will be able to deal with ‘N’ number of forces representing different attractive entities for Cancer cell membrane coated Nanoparticles in hyperspace.

## Data availability statement

The original contributions presented in the study are included in the article/**Supplementary Material**. Further inquiries can be directed to the corresponding authors.

## Author contributions

SaK developed the probabilistic model, and its assumptions, SaK and MK implemented the model in python, SS and SA performed necessary validation studies, MA and KA-M determined binding scores. ShK developed tailor designed nanoparticles for validation studies. All authors contributed to the article and approved the submitted version.
