# Supplementary figures and images for: Directional preference for glioblastoma cancer cell membrane encapsulated nanoparticle population: A probabilistic approach for cancer therapeutics
Source: Front Immunol. 2023 Mar 29;14:1162213. doi: 10.3389/fimmu.2023.1162213 (PMC10090548; doi:10.3389/fimmu.2023.1162213)

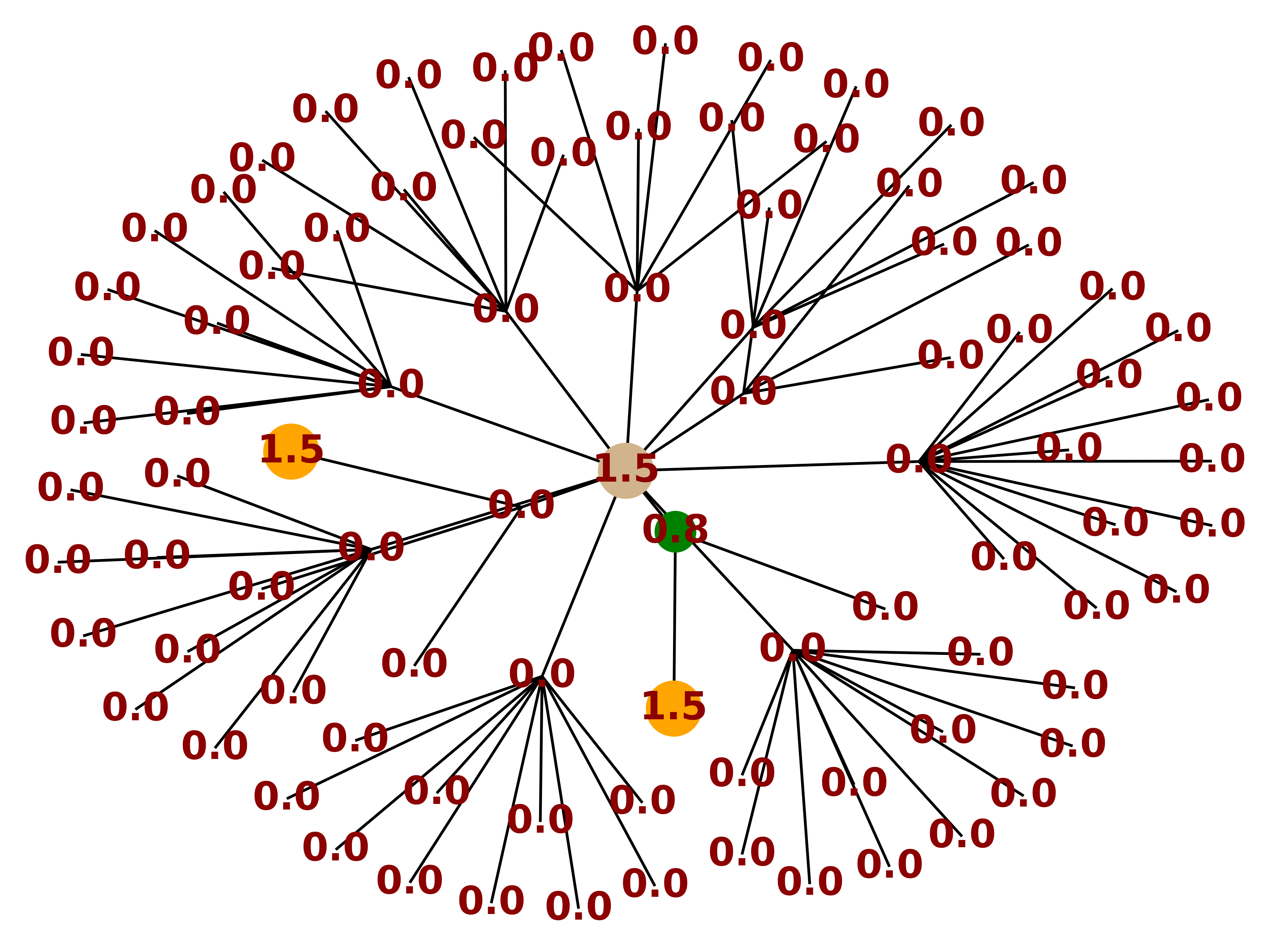

Supplement: Supplementary file 1 [file Image_1.png]

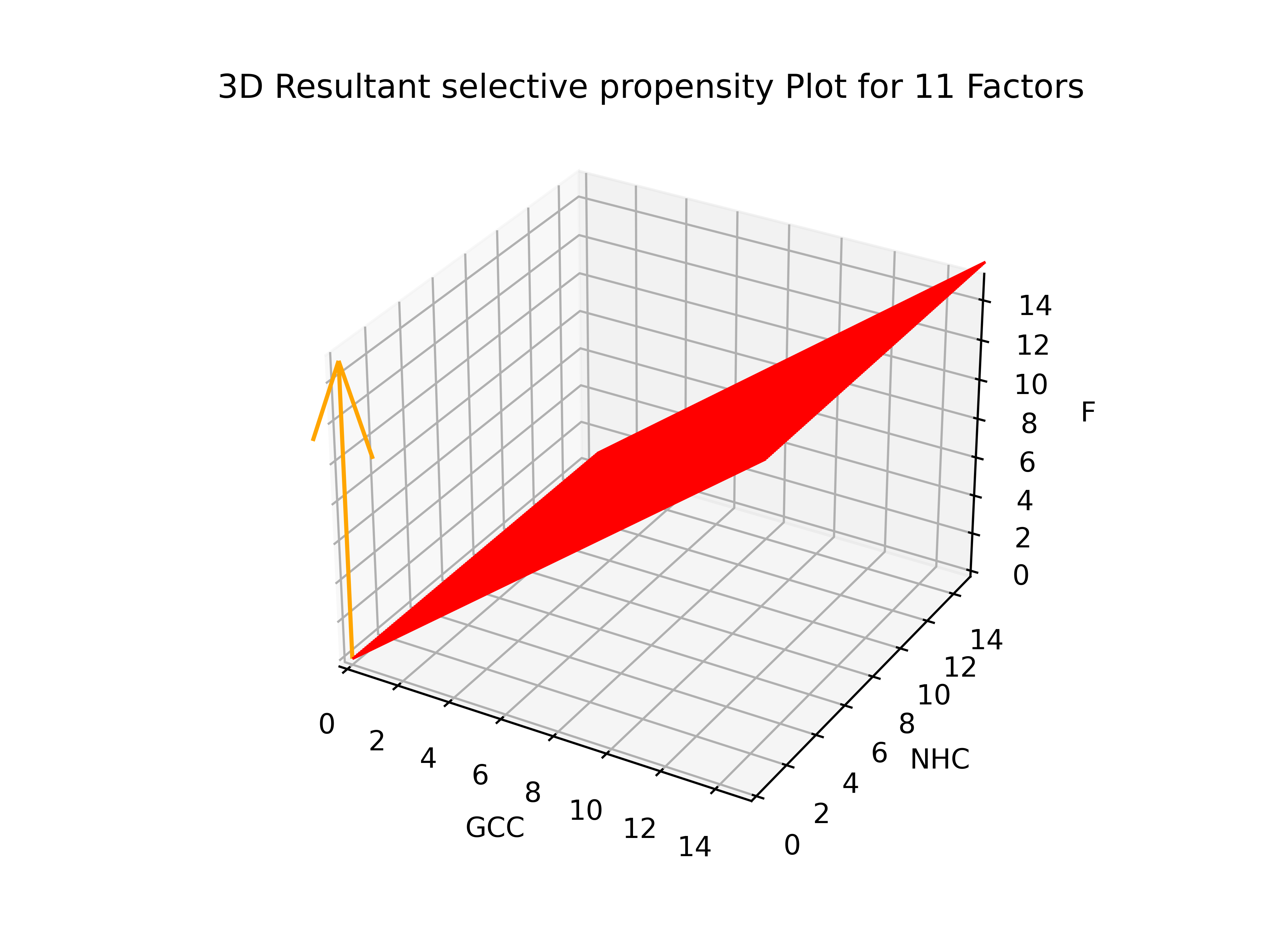

Supplement: Supplementary file 2 [file Image_2.png]
